# Climate change and mental health: an urgent call for strategic action from nursing

**DOI:** 10.1590/0034-7167-2025-0604

**Published:** 2026-08-07

**Authors:** Ítalo Arão Pereira Ribeiro, Iel Marciano de Moraes, Ana Lívia Castelo Branco de Oliveira, José Claudio Garcia Lira, Giovana Galvão Tavares, Márcia Astrês Fernandes

**Affiliations:** I Universidade Federal de Mato Grosso do Sul. Coxim, Mato Grosso do Sul, Brazil; II Universidade Evangélica de Goiás, Programa de Pós-Graduação em Sociedade, Tecnologia e Meio Ambiente. Anápolis, Goiás, Brazil; III Universidade Federal do Piauí. Floriano, Piauí, Brazil; IV Universidade Federal do Piauí. Teresina, Piauí, Brazil

The need to understand grief in the current global context, marked by crises and worldwide challenges such as pandemics, wars, and social violence, highlights the relevance of this study, since the analysis of grief, the adaptation process experienced by bereaved individuals, coping strategies, and the moment when professional help should be considered are important for mental health and collective subjective well-being. Several factors influence the elaboration and experience of grief, among which the circumstances surrounding the loss experienced by the individual stand out. Understanding aspects related to the process of loss, death, and dying is important for comprehending grief^(1-4)^.

Grief may be defined as the feeling triggered by a significant loss of the bond between a person and their object. Characterized by sadness, focus on absence, and lack of motivation, grief is not limited to death, but also includes symbolic or real losses throughout an individual’s development^(2,3)^.

Experiencing a definitive rupture may be considered traumatic, especially due to the circumstances of the loss and the importance of the absence of the lost bond^(1,4)^. Lacan^(2)^ describes grief as something to be overcome in an intersubjective manner, considering that external factors, such as emotional relationships and the way individuals live in society, interfere with the overcoming process.

Considering that grief represents the pain of loss, the process that follows it represents the transformation of the bereaved individual through the re-signification of life itself. This period is understood as a process of elaboration and adaptation. Freud^(3)^ considers that time overcomes pain and that grief, as a natural process, should not be interfered with. However, when it does not evolve naturally, it becomes characterized as melancholia, a pathological state of reaction to loss.

For grief to be overcome, it is important to re-signify what has been lost. It is not enough for the object or person to disappear for them to be forgotten; slow and often painful psychic work is necessary^(3)^. Loss may cause pain, but it may also promote growth and emotional strengthening. Resilience and the capacity for adaptation are fundamental in this process^(4)^.

As a particular process, grief involves emotional and psychological aspects, such as the ability to reconnect with the external world, fill the void left by the loss, and re-signify the present and the future. It also involves social aspects related to interaction with the external world. Thus, when grief is overcome, the person’s inner world is transformed and the loss is re-signified^(2,4)^.

Although the process is singular, there are moments when the need for professional support may become evident, especially from mental healthcare professionals, such as nurses^(1)^. Behavioral, cognitive, and emotional reactions vary and may include sadness, anger, guilt, hopelessness, and, in some cases, relief^(1)^.

When grief is not finite and there is a feeling that the lost object also represents the death of part of the bereaved individual, seeking professional help becomes essential. Healthcare professionals need to be prepared to identify grief symptoms, understand the grieving process, and provide support in coping with and alleviating suffering^(3-4)^.

Nurses have the mission of providing comprehensive care, offering emotional, social, and physical support, in addition to comfort and attention. They experience loss in their daily practice and are responsible for accompanying patients and bereaved individuals, playing an essential role in this process^(1)^. Therefore, there is a constant need for professional qualification in order to provide effective care without allowing work itself to become a factor contributing to illness. It may be concluded that understanding grief in psychoanalysis and discussing the overcoming process help these healthcare professionals develop proposals for follow-up care and support for bereaved individuals.

This discussion promotes greater awareness regarding grief symptoms and coping processes, ranging from those considered normal to those that may represent pathological conditions. Psychoanalysis does not understand grief merely as an event, but also as a way of subjectivizing the pain of loss, which may become constant. The deeper the discussion, the greater the understanding of the processes of suffering and adaptation.

Finally, the importance of nurses and other healthcare professionals remaining updated and adequately trained for this type of care is reinforced, as well as the need for them to receive social and psychological support in order to provide humanized welcoming care.
