# ERRATUM: Climate change and mental health: an urgent call for strategic action from nursing

**DOI:** 10.1590/0034-7167.202679e0520250604

**Published:** 2026-10-05

In the article “Climate change and mental health: an urgent call for strategic action from nursing”, with DOI number: https://doi.org/10.1590/0034-7167.20267904, published in Revista Brasileira de Enfermagem, 2026;79:e7904:

Where it read:

https://doi.org/10.1590/0034-7167.20267904

Rev Bras Enferm. 2026;79:e7904

It reads:

https://doi.org/10.1590/0034-7167-2025-0604

Rev Bras Enferm. 2026;79:e20250604

Prof. PhD Dulce Barbosa

**Editor in Chief**

