# Supplementary material for: Climate change and mental health: an urgent call for strategic action from nursing
Source: Rev Bras Enferm. 2026 Aug 7;79:e20250604. doi: 10.1590/0034-7167-2025-0604 (PMC13450829; doi:10.1590/0034-7167-2025-0604)
Supplement: Translation

# Translation: Mudanças climáticas e saúde mental: um chamado urgente à atuação estratégica da enfermagem

**DOI:** 10.1590/0034-7167-2025-0604pt

**Authors:** Ítalo Arão Pereira Ribeiro, Iel Marciano de Moraes, Ana Lívia Castelo Branco de Oliveira, José Claudio Garcia Lira, Giovana Galvão Tavares, Márcia Astrês Fernandes

**Affiliations:** I Universidade Federal de Mato Grosso do Sul. Coxim, Mato Grosso do Sul, Brasil; II Universidade Evangélica de Goiás, Programa de Pós-Graduação em Sociedade, Tecnologia e Meio Ambiente. Anápolis, Goiás, Brasil; III Universidade Federal do Piauí. Floriano, Piauí, Brasil; IV Universidade Federal do Piauí. Teresina, Piauí, Brasil

As mudanças climáticas se consolidaram como uma das ameaças mais sérias à saúde humana no século XXI, com impactos significativos não apenas ao meio ambiente, mas também aos determinantes sociais de saúde e ao bem-estar psíquico das pessoas. O aumento na frequência e intensidade de fenômenos climáticos extremos como inundações, secas, ondas de calor, incêndios florestais e ciclones tem gerado perdas materiais, deslocamentos forçados, insegurança alimentar e hídrica, (re)surgimento de pandemias e um aumento de doenças transmitidas por mosquitos, causando profundas sequelas socioemocionais. Diante desse panorama, torna-se cada vez mais urgente reconhecer e discutir os efeitos dessas mudanças sobre a saúde mental, especialmente em populações vulnerabilizadas^(1)^.

Evidências têm apontado para uma correlação crescente entre os eventos climáticos extremos e o surgimento ou agravamento de transtornos mentais como ansiedade, depressão, transtorno de estresse pós-traumático, insônia e uso problemático de substâncias psicoativas^(1)^. Relacionado a isso, desordens emocionais pelo medo do rompimento de vínculos afetivos, instabilidade econômica, perda de bens materiais, moradia ou entes queridos têm provocado um aumento expressivo na ocorrência de estresse, isolamento social, experiências traumáticas e desesperança^(2)^.

Esse cenário tem sido descrito como ecoansiedade ou ansiedade climática. Ecoansiedade é um fenômeno psicossocial caracterizado por medo persistente diante das mudanças climáticas e das incertezas relacionadas ao futuro do planeta, ou ainda à solastalgia, termo que expressa o sofrimento emocional decorrente da degradação ambiental de lugares com forte valor afetivo^(2)^. Esses fenômenos tendem a afetar, de forma mais intensa, crianças, adolescentes e populações expostas às iniquidades sociais e ao racismo ambiental, especialmente aquelas cujas identidades, modos de vida e vínculos culturais estão intrinsecamente ligados ao ambiente natural, ampliando desigualdades em saúde e vulnerabilidades psicossociais.

Nesse contexto, o papel da enfermagem torna-se estratégico. A capilaridade da profissão nos diferentes níveis de atenção à saúde, aliada ao expressivo contingente de profissionais e ao enraizamento territorial, favorece vínculos diretos e contínuos com as comunidades. Não raro, enfermeiras(os) são as(os) primeiras(os) a acolher manifestações de sofrimento psíquico da população, sobretudo em territórios marcados pela vulnerabilidade social. É imperioso reconhecer e abordar precocemente sinais de sofrimento emocional relacionado às mudanças climáticas, oferecer escuta qualificada e acolhimento empático, promover encaminhamentos assertivos, e desenvolver ações de fortalecimento da saúde mental e da resiliência comunitária^(1)^.

Além do cuidado individual, a prática de enfermagem precisa incorporar, de maneira intencional, os princípios da justiça climática e da equidade em saúde, reconhecendo que os impactos da crise climática são desiguais e recaem com maior severidade sobre grupos historicamente marginalizados, isto é, populações afrodescendentes, indígenas, ribeirinhas, pessoas em situação de rua, migrantes ambientais e favelados. Esses grupos vivenciam barreiras estruturais para acessar os serviços de saúde mental, enfrentando múltiplas barreiras que se potencializam diante da ausência de políticas públicas sensíveis ao contexto socioambiental, o que pode ampliar injustiças e sofrimentos evitáveis.

Diante disso, impõe-se revisão nos currículos de enfermagem, em todos os níveis - técnico, de graduação e pós-graduação - para incorporar conteúdos sobre mudanças climáticas, saúde mental, emergências ambientais, saúde planetária e justiça climática. A formação deve contemplar estratégias de educação em saúde planetária para qualificar enfermeiras(os) na gestão do sofrimento psíquico em situações de desastres, na avaliação de riscos socioambientais, na comunicação de riscos à população, no cuidado culturalmente sensível e na aplicação de práticas baseadas em evidências orientadas para a crise climática como um desafio de saúde pública global^(3,4)^.

É igualmente necessário ampliar a presença e a influência da enfermagem em espaços onde decisões são tomadas. A participação ativa da categoria na formulação de micropolíticas e políticas públicas em conselhos de saúde, comitês de gestão de riscos, fóruns intersetoriais e movimentos socioambientais é indispensável para integrar saúde, meio ambiente, assistência social e educação sob um olhar de integralidade. Essa inserção possibilita um deslocamento da perspectiva tradicional de “enfermagem global” para uma enfermagem planetária alinhada aos princípios da saúde planetária e guiada por uma prática ética, humanística e comprometida com a sustentabilidade da vida.

Mais do que nunca, a enfermagem é chamada a ocupar seu papel de liderança ética e social diante da crise climática - não apenas como prestadora de cuidado, mas como agente transformador de realidades e defensora da vida em todas as suas formas.

# Translation: Cambio climático y salud mental: un llamado urgente a la acción estratégica de la enfermería

**DOI:** 10.1590/0034-7167-2025-0604es

**Authors:** Ítalo Arão Pereira Ribeiro, Iel Marciano de Moraes, Ana Lívia Castelo Branco de Oliveira, José Claudio Garcia Lira, Giovana Galvão Tavares, Márcia Astrês Fernandes

**Affiliations:** I Universidade Federal de Mato Grosso do Sul. Coxim, Mato Grosso do Sul, Brasil; II Universidade Evangélica de Goiás, Programa de Pós-Graduação em Sociedade, Tecnologia e Meio Ambiente. Anápolis, Goiás, Brasil; III Universidade Federal do Piauí. Floriano, Piauí, Brasil; IV Universidade Federal do Piauí. Teresina, Piauí, Brasil

El cambio climático se ha convertido en una de las amenazas más graves para la salud humana en el siglo XXI, con impactos significativos no sólo en el medio ambiente sino también en los determinantes sociales de la salud y el bienestar mental de las personas. La mayor frecuencia e intensidad de fenómenos meteorológicos extremos, como inundaciones, sequías, olas de calor, incendios forestales y ciclones, ha provocado pérdidas materiales, desplazamientos forzados, inseguridad alimentaria y hídrica, la (re)emergencia de pandemias y un aumento de las enfermedades transmitidas por mosquitos, lo que ha provocado profundas consecuencias socioemocionales. Ante este escenario, se hace cada vez más urgente reconocer y discutir los efectos de estos cambios en la salud mental, especialmente en las poblaciones vulnerables^(1)^.

La evidencia ha señalado una correlación creciente entre los fenómenos climáticos extremos y la aparición o empeoramiento de trastornos de salud mental como ansiedad, depresión, trastorno de estrés postraumático, insomnio y uso problemático de sustancias psicoactivas^(1)^. En relación a esto, los trastornos emocionales derivados del miedo a la ruptura de vínculos afectivos, la inestabilidad económica, la pérdida de bienes materiales, de vivienda o de seres queridos han provocado un aumento significativo en la aparición de estrés, aislamiento social, experiencias traumáticas y desesperanza^(2)^.

Este escenario se ha descrito como ecoansiedad o ansiedad climática. La ecoansiedad es un fenómeno psicosocial caracterizado por el miedo persistente ante el cambio climático y las incertidumbres relacionadas con el futuro del planeta, o incluso solastalgia, término que expresa el sufrimiento emocional derivado de la degradación ambiental de lugares con un fuerte valor afectivo^(2)^. Estos fenómenos tienden a afectar con mayor intensidad a niños, adolescentes y poblaciones expuestas a inequidades sociales y racismo ambiental, especialmente a aquellos cuyas identidades, estilos de vida y vínculos culturales están intrínsecamente ligados al entorno natural, amplificando así las desigualdades en salud y las vulnerabilidades psicosociales.

En este contexto, el papel de la enfermería se vuelve estratégico. Su alcance en los diferentes niveles de atención sanitaria, sumado al considerable número de profesionales y su fuerte arraigo territorial, fomenta vínculos directos y continuos con las comunidades. No es raro que las(los) enfermeras(os) sean las(los) primeras(os) en abordar las manifestaciones de malestar psicológico en la población, especialmente en zonas de vulnerabilidad social. Es imperativo reconocer y abordar los signos tempranos de angustia emocional relacionada con el cambio climático, ofrecer escucha calificada y apoyo empático, promover referencias asertivas y desarrollar acciones para fortalecer la salud mental y la resiliencia comunitaria^(1)^.

Más allá del cuidado individual, la práctica de enfermería debe incorporar intencionalmente los principios de justicia climática y equidad en salud, reconociendo que los impactos de la crisis climática son desiguales y recaen más severamente sobre los grupos históricamente marginados, a saber, las poblaciones afrodescendientes, indígenas, ribereñas, sin hogar, migrantes ambientales y de favelas. Estos grupos experimentan barreras estructurales para acceder a los servicios de salud mental, enfrentando múltiples obstáculos que se agravan por la ausencia de políticas públicas sensibles al contexto socioambiental, lo que puede amplificar injusticias y sufrimientos evitables.

Por lo tanto, es necesaria una revisión de los planes de estudio de enfermería en todos los niveles (técnico, de pregrado y de posgrado) para incorporar contenidos sobre cambio climático, salud mental, emergencias ambientales, salud planetaria y justicia climática. La formación debe incluir estrategias de educación en salud planetaria para calificar enfermeras(os) en el manejo del distrés psicológico en situaciones de desastre, la evaluación de riesgos socioambientales, la comunicación de riesgos a la población, la prestación de atención culturalmente sensible y la aplicación de prácticas basadas en evidencia orientadas a la crisis climática como un desafío de salud pública global^(3,4)^.

Es igualmente necesario ampliar la presencia e influencia de la enfermería en los espacios de toma de decisiones. La participación activa de la profesión en la formulación de micropolíticas y políticas públicas en consejos de salud, comités de gestión de riesgos, foros intersectoriales y movimientos socioambientales es indispensable para integrar la salud, el medio ambiente, la asistencia social y la educación desde una perspectiva holística. Esta inclusión permite un cambio desde la perspectiva tradicional de la “enfermería global” a una enfermería planetaria alineada con los principios de la salud planetaria y guiada por una práctica ética, humanista y comprometida con la sostenibilidad de la vida.

Más que nunca, la enfermería está llamada a asumir su papel de liderazgo ético y social frente a la crisis climática, no sólo como proveedora de cuidados, sino como agente de cambio y defensora de la vida en todas sus formas.
