# Supplementary material for: ERRATUM: Climate change and mental health: an urgent call for strategic action from nursing
Source: Rev Bras Enferm. 2026 Oct 5;79:e2026e0520250604. doi: 10.1590/0034-7167.202679e0520250604 (PMC13645328; doi:10.1590/0034-7167.202679e0520250604)
Supplement: Related texts

# Correction: ERRATA: Enfermeiros e o luto: uma análise sob a perspectiva da psicanálise

**DOI:** 10.1590/0034-7167.202679e0520250604pt

No artigo “Enfermeiros e o luto: uma análise sob a perspectiva da psicanálise”, com número DOI: https://doi.org/10.1590/0034-7167.20267904pt, publicado no periódico Revista Brasileira de Enfermagem, 2026;79:e7904:

No Título, onde se lê:

**Enfermeiros e o luto: uma análise sob a perspectiva da psicanálise**

Lê-se:

**Mudanças climáticas e saúde mental: um chamado urgente à atuação estratégica da enfermagem**

Onde se lê:

https://doi.org/10.1590/0034-7167.20267904pt

Rev Bras Enferm. 2026;79:e7904

Leia-se:

https://doi.org/10.1590/0034-7167-2025-0604pt

Rev Bras Enferm. 2026;79:e20250604

Profa. Dra. Dulce Barbosa

*Editora-Chefe*

# Correction: ERRATA: Cambio climático y salud mental: un llamado urgente a la acción estratégica de la enfermería

**DOI:** 10.1590/0034-7167.202679e0520250604es

En el artículo “Cambio climático y salud mental: un llamado urgente a la acción estratégica de la enfermería”, con número DOI: https://doi.org/10.1590/0034-7167.20267904es, publicado en la Revista Brasileira de Enfermagem, 2026;79:e7904:

Donde decía:

https://doi.org/10.1590/0034-7167.20267904es

Rev Bras Enferm. 2026;79:e7904

Dice:

https://doi.org/10.1590/0034-7167-2025-0604es

Rev Bras Enferm. 2026;79:e20250604

Prof. Dra. Dulce Barbosa

*Editora en jefe*
